# A 1:1 co-crystal of the herbicide triflusulfuron-methyl and its degradation product triazine amine

**DOI:** 10.1107/S1600536811031631

**Published:** 2011-08-11

**Authors:** Kurt Mereiter

**Affiliations:** a Institute of Chemical Technologies and Analytics, Vienna University of Technology, Getreidemarkt 9/164SC, A-1060 Vienna, Austria

## Abstract

The herbicide triflusulfuron-methyl (systematic name: methyl 2-{[4-dimethyl­amino-6-(2,2,2-trifluoro­eth­oxy)-1,3,5-triazin-2-yl]carbamoylsulfamo­yl}-3-methyl­benzoate) and its degradation product triazine amine [systematic name: 2-amino-4-dimethyl­amino-6-(2,2,2-trifluoro­eth­oxy)-1,3,5-triazine] form a triclinic 1:1 co-crystal of the title compound, C_7_H_10_F_3_N_5_O·C_17_H_19_F_3_N_6_O_6_S, in which its two components are connected *via* a pair of complementary N—H⋯N hydrogen bonds, similar to the monoclinic crystal structure of the parent compound triflusulfuron-methyl [Mereiter (2011 ▶). *Acta Cryst.* E**67**, o1778–o1779] in which a pair of mol­ecules related by a twofold axis are linked by two N—H⋯N bonds. The triflusulfuron-methyl mol­ecules of both crystal structures are similar in geometric parameters and conformation, which is due to stiffening by a short intra­molecular N—H⋯N bond [N⋯N = 2.620 (4) Å] and an intra­molecular dipole–dipole inter­action between the sulfamide and the carboxyl moieties, with O_s_⋯C_c_ = 2.802 (5) Å and O_c_⋯N_s_ = 2.846 (4) Å. Inter­molecular N—H⋯O hydrogen bonds and slipped π–π stacking inter­actions between the diamino­triazine moieties [perpendicular distances of 3.25 Å within hydrogen-bonded tetra­mers and 3.27 Å between adjacent tetra­mers] link the two constituents of the co-crystal into columns parallel to the *a* axis. An intra­molecular C—H⋯O hydrogen bond occurs in the triflusulfuron-methyl mol­ecule and inter­molecular C—H⋯O inter­actions between triflusulfuron-methyl mol­ecules occur in the crystal structure. In the triflusulfuron-methyl molecule the dihedral angle between the least-squares planes of the two rings is 75.8 (1)°. In the triazine molecule, the CF_3_ group is partly orientationally disordered.

## Related literature

For the crystal structure of the herbicide triflusulfuron-methyl, see: Mereiter (2011 ▶). For information on the synthesis and herbicidal properties of triflusulfuron-methyl, see: Moon (1989 ▶); Peeples *et al.* (1991 ▶); Wittenbach *et al.* (1994 ▶). For general information on the herbicidal properties of triflusulfuron-methyl and its degradation product triazine amine, see: EFSA (2008 ▶).
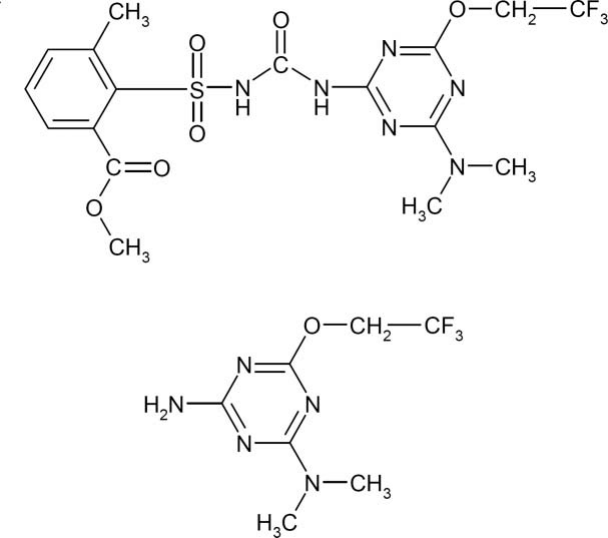


## Experimental

### 

#### Crystal data

C_7_H_10_F_3_N_5_O·C_17_H_19_F_3_N_6_O_6_S
*M* *_r_* = 729.64
Monoclinic, 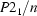

*a* = 9.0388 (18) Å
*b* = 12.120 (2) Å
*c* = 27.820 (5) Å
β = 91.883 (3)°
*V* = 3046.0 (10) Å^3^
*Z* = 4
Mo *K*α radiation
μ = 0.21 mm^−1^
*T* = 100 K
0.42 × 0.03 × 0.03 mm

#### Data collection

Bruker KAPPA APEXII CCD diffractometer
Absorption correction: multi-scan (*SADABS*; Bruker, 2008 ▶) *T*_min_ = 0.87, *T*_max_ = 0.99
29573 measured reflections
5239 independent reflections
3575 reflections with *I* > 2σ(*I*)
*R*_int_ = 0.085

#### Refinement

*R*[*F* ^2^ > 2σ(*F* ^2^)] = 0.059
*wR*(*F* ^2^) = 0.152
*S* = 1.04
5239 reflections
461 parameters
31 restraints
H-atom parameters constrained
Δρ_max_ = 0.36 e Å^−3^
Δρ_min_ = −0.45 e Å^−3^

### 

Data collection: *APEX2* (Bruker, 2008 ▶); cell refinement: *SAINT* (Bruker, 2008 ▶); data reduction: *SAINT*, *SADABS* and *XPREP* (Bruker, 2008 ▶); program(s) used to solve structure: *SHELXS97* (Sheldrick, 2008 ▶); program(s) used to refine structure: *SHELXL97* (Sheldrick, 2008 ▶); molecular graphics: *Mercury* (Macrae *et al.*, 2006 ▶); software used to prepare material for publication: *PLATON* (Spek, 2009 ▶) and *publCIF* (Westrip, 2010 ▶).

## Supplementary Material

Crystal structure: contains datablock(s) I, global. DOI: 10.1107/S1600536811031631/pk2336sup1.cif

Structure factors: contains datablock(s) I. DOI: 10.1107/S1600536811031631/pk2336Isup2.hkl

Supplementary material file. DOI: 10.1107/S1600536811031631/pk2336Isup3.cml

Additional supplementary materials:  crystallographic information; 3D view; checkCIF report

## Figures and Tables

**Table 1:** Hydrogen-bond geometry (Å, °)

*D*—H⋯*A*	*D*—H	H⋯*A*	*D*⋯*A*	*D*—H⋯*A*
N1—H1*N*⋯N5	0.88	1.94	2.620 (4)	133
N2—H2*N*⋯N8	0.88	2.20	3.080 (4)	176
N7—H7*NA*⋯N3	0.88	2.11	2.989 (5)	174
N7—H7*NB*⋯O3^i^	0.88	2.13	2.996 (4)	167
C5—H5⋯O2^ii^	0.95	2.57	3.434 (5)	152
C8—H8*A*⋯O2^iii^	0.98	2.43	3.398 (5)	170
C9—H9*A*⋯O5	0.98	2.40	3.210 (5)	140
C16—H16*B*⋯O5^i^	0.99	2.47	3.373 (5)	152

Symmetry codes: (i) 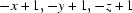; (ii) 
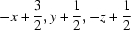
; (iii) 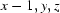.

**Fig. 1.**
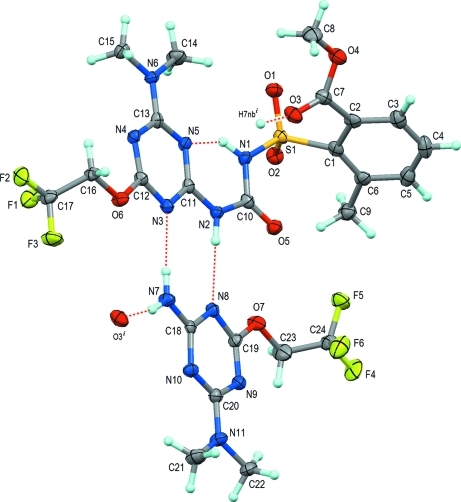
Asymmetric unit of (I) with displacement ellipsoids for the non-hydrogen atoms drawn at the 50% probability level. Hydrogen bonds are drawn as dashed red lines. Symmetry code i: 1 - x, 1 - y, 1 - z. Intramolecular interactions are N1···N5 = 2.620 (4) Å, O1···C7 = 2.802 (5) Å, and O3···N1 = 2.846 (4) Å. Here and in all subsequent Figures only the dominant set of sites of the disordered C24F3 group is shown.

**Fig. 2.**
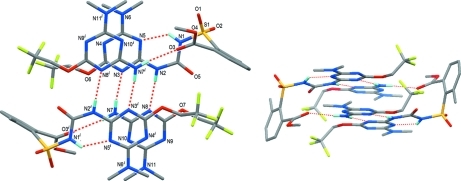
Top and side view of the cyclically hydrogen bonded tetramer of two TFS and two TA molecules in (I). C-bonded hydrogen atoms omitted for clarity.

**Fig. 3.**
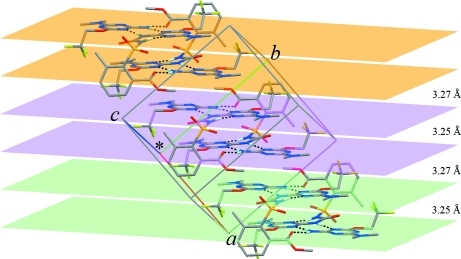
π-π-Stacking of the hydrogen bonded tetramers shown in Fig. 2 to form a column parallel to the a-axis. The coloured planes are fitted through each two triazine rings. Plane-to-plane distances are given on the right. C-bonded H-atoms omitted for clarity. Origin of the unit cell marked by an asterisk.

**Fig. 4.**
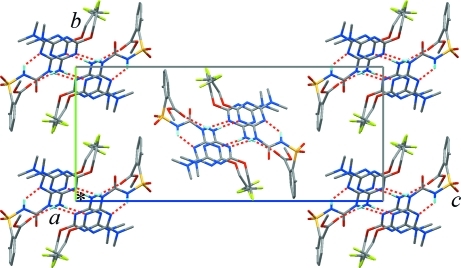
Packing diagram of (I) in a view down the a-axis. C-bonded H-atoms omitted for clarity. Origin of the unit cell marked by an asterisk.

## supplementary crystallographic information

### Comment

The crystal structure of the triazinylsulfonylurea herbicide triflusulfuron-methyl, C_17_H_19_F_3_N_6_O_6_S, marketed under the trade names UpBeet, Debut, and Safari (originator DuPont^TM^; Moon, 1989; Peeples *et al.*, 1991; Wittenbach *et al.*, 1994) for crop protection of sugar beet and fodder beet, was recently reported (Mereiter, 2011). The compound crystallizes in a monoclinic lattice of space group *C*2*/c* with *a* = 16.7107 (11) Å, *b* = 15.6406 (11) Å, *c* = 17.1875 (12) Å, *β* = 107.035 (1)°, *V* = 4295.1 (5) Å^3^, and *Z* = 8 at *T* = 100 K. This crystal form is subsequently denoted CMTFS whereas the chemical entity triflusulfuron-methyl is denoted TFS. Triflusulfuron-methyl degrades in aqueous solutions by hydrolytic cleavage of the sulfonylurea bridge yielding CO_2_, CH_3_OH, methyl-saccharine [systematic name: 7-methyl-1,2-benzisothiazol-3(2*H*)-one-1,1-dioxide, C_8_H_7_NO_3_S] and triazine amine [2-amino-4-(dimethylamino)-6-(2,2,2-trifluoroethoxy)-1,3,5-triazine, C_7_H_10_F_3_N_5_O], denoted TA, as the primary products (EFSA, 2008). TA is a relevant impurity of technical TFS and should not exceed 0.6% for crop protection purposes (EFSA, 2008). During crystallization experiments of TFS (Mereiter, 2011), a new crystal species of triclinic symmetry and space group *P*1 was obtained the crystal structure of which is reported here. The title compound, (I), is a 1:1 cocrystal of TFS and TA as shown in Scheme 1 and Fig. 1. In this crystal structure, the TFS molecule exhibits geometric features similar to those in CMTFS (Mereiter, 2011). These characteristics are: (i) an approximately planar system formed by the diaminotriazine moiety and the adjacent atoms N2, C10, O5, N1, S1, O1, O6, and C16 (r.m.s. deviation from planarity 0.139 Å); (ii) a flat 2-methylphenyl group inclined to (i) at an angle of 75.8 (1)°; (iii) a flat carboxymethyl group inclined to (ii) by 47.9 (2)°; (iv) a short intramolecular hydrogen bond N1—H1n···N5, N1···N5 = 2.620 (4) Å (Table 1); and (v) a striking intramolecular dipole-dipole interaction between the sulfamide and the carboxylate moiety with the short distances O1···C7 = 2.802 (5) Å and O3···N1 = 2.846 (4) Å, which are important for the conformation of the molecule and its chemical reactivity. The corresponding dimensions in CMTFS are 75.26 (2)° and 48.07 (6)° for the interplanar angles and 2.641 (1), 2.800 (1), and 2.835 (1) Å, respectively, for the distances (Mereiter, 2011). At variance with CMTFS the carbon atom C17 of the CF_3_ group in (I) is not coplanar with the triazine ring but twisted from it and has a torsion angle of C12—O6—C16—C17 = 127.5 (4)°. The corresponding angle in CMTFS is 176.1 (1)°. The TA molecule in (I) has dimensions similar to the corresponding fragment in the TFS molecule. Here too, is the carbon atom C24 of the CF_3_ group not coplanar with the rest of the molecule. This CF_3_ group is disordered over two sets of sites (see section refinement), of which the dominant set has a torsion angle of C19—O7—C23—C24 = -110.7 (4)° and the subordinate set an angle of C19—O7—C23—C24' = -141.0 (8)°. The mutual arrangement of the two components of (I) in the asymmetric unit and their connection by a pair of complementary N—H···N hydrogen bonds, N2—H2n···N8 (N···N = 3.080 (4) Å) and N7—H7na···N3 (N···N = 2.989 (5) Å) is visualized in Fig. 1. The two triazine rings in Fig. 1 deviate only by 0.038 Å from a common l.s. plane. This pair of molecules is linked with another centrosymmetric pair *via* two hydrogen bonds N7—H7nb···O3^i^ (N···O = 2.996 (4) Å, O3 is a carboxyl oxygen) to form a finite cyclically hydrogen bonded tetramer of two TFS and two TA molecules shown in Fig. 2. The tetramers are stacked one above the other along the *a*-axis to form infinite columns, as shown in Fig. 3. Coherence within and between the hydrogen-bonded tetramers along the *a*-axis is provided by slipped π-π-stacking interactions with perpendicular distances of 3.25 Å within hydrogen bonded tetramers and 3.27 Å between adjacent tetramers. These distances refer to the combined mean plane of the two triazine rings in TFS and TA mentioned above; the planes are approximately parallel to either (111) or to its symmetry equivalent (111). A view of the resulting crystal structure seen along the *a*-axis, *i.e.* along the π-π-stacked columns, is given in Fig. 4. Perpendicular to the *a*-axis the columns are held together by van der Waals forces and several unremarkable C—H···O interactions (Table 1). The *a*-axis is also the needle axis of the crystals. The hydrogen bonded dimer TFS and TA shown in Fig. 1 resembles closely the situation in CMTFS, where a pair of TFS molecules related by a twofold axis is linked *via* two complementary N—H···N bonds measuring N···N = 2.900 (1) Å (Mereiter, 2011). However, the further spatial arrangement of the molecules in CMTFS differs significantly from (I).

Concluding remark: Cocrystal formation of bioactive compounds is a field of research nowadays very actively pursued with the goal of achieving new solids with superior properties like crystallization propensity, improved chemical stability, solubility, dissolution rate, *etc.*. In case of the title compound the cocrystal formation is interesting, but is not desirable on following grounds: The triazine amine as one of the two components of the cocrystal of (I) has unwanted biocidal properties, and the cocrystal formation may probably impede the purification of triazine amine contaminated triflusulfuron-methyl by industrial batch crystallization.

### Experimental

In search of new crystal polymorphs a sample of technical triflusulfuron-methyl contaminated by some triazine amine (2-amino-4-(dimethylamino)-6-(2,2,2-trifluoroethoxy)-1,3,5-triazine) was dissolved in hot ethanol and boiled under reflux for 10 minutes. The solution was then cooled to room temperature and the solvent slowly evaporated within two days. Thin colorless needles of the title compound accompanied by larger crystals of triflusulfuron-methyl were obtained.

### Refinement

H atoms were located in a difference Fourier map, placed in calculated positions (N—H = 0.88 Å, C—H = 0.95 - 0.99 Å) and thereafter treated as riding. A torsional parameter was refined for each methyl group. *U*_iso_(H) = 1.2*U*_eq_(C,*N*) for CH, CH_2_, NH and NH_2_ groups; *U*_iso_(H) = 1.5*U*_eq_(C) for CH_3_ groups. The CF_3_ group of the triazine amine molecule (C24, F4, F5, and F6) is disordered over two sets of sites in a 0.790 (5)/0.210 (5) ratio. In the final refinement both sets of sites were stabilized with three SADI 0.03 restraints of program *SHELXL97* for the bonds C23—C24/C23—C24', for the C—F bonds, and for the internal F—F distances. The atoms of the subordinate set of sites were refined with isotropic displacement parameters fixed at *U*_iso_(C) = 0.031 and *U*_iso_(F) = 0.046 Å^2^ corresponding to the *U*_eq_ values of the corresponding dominant sites (mean value of F4, F5, F6 for F4', F5', F6').

### Crystal data

**Table:**

C_7_H_10_F_3_N_5_O·C_17_H_19_F_3_N_6_O_6_S	*F*(000) = 1504
*M**_r_* = 729.64	*D*_x_ = 1.591 Mg m^−^^3^
Monoclinic, *P*2_1_/*n*	Mo *K*α radiation, λ = 0.71073 Å
Hall symbol: -P 2yn	Cell parameters from 3426 reflections
*a* = 9.0388 (18) Å	θ = 2.8–22.4°
*b* = 12.120 (2) Å	µ = 0.21 mm^−^^1^
*c* = 27.820 (5) Å	*T* = 100 K
β = 91.883 (3)°	Needle, colourless
*V* = 3046.0 (10) Å^3^	0.42 × 0.03 × 0.03 mm
*Z* = 4	

### Data collection

**Table:**

Bruker KAPPA APEXII CCD diffractometer	5239 independent reflections
Radiation source: fine-focus sealed tube	3575 reflections with *I* > 2σ(*I*)
graphite	*R*_int_ = 0.085
φ and ω scans	θ_max_ = 24.9°, θ_min_ = 2.9°
Absorption correction: multi-scan (*SADABS*; Bruker, 2008)	*h* = −10→10
*T*_min_ = 0.87, *T*_max_ = 0.99	*k* = −14→14
29573 measured reflections	*l* = −32→32

### Refinement

**Table:**

Refinement on *F*^2^	Primary atom site location: structure-invariant direct methods
Least-squares matrix: full	Secondary atom site location: difference Fourier map
*R*[*F*^2^ > 2σ(*F*^2^)] = 0.059	Hydrogen site location: inferred from neighbouring sites
*wR*(*F*^2^) = 0.152	H-atom parameters constrained
*S* = 1.04	*w* = 1/[σ^2^(*F*_o_^2^) + (0.0577*P*)^2^ + 6.8112*P*] where *P* = (*F*_o_^2^ + 2*F*_c_^2^)/3
5239 reflections	(Δ/σ)_max_ < 0.001
461 parameters	Δρ_max_ = 0.36 e Å^−^^3^
31 restraints	Δρ_min_ = −0.45 e Å^−^^3^

### Special details

**Table:**

Geometry. All e.s.d.'s (except the e.s.d. in the dihedral angle between two l.s. planes) are estimated using the full covariance matrix. The cell e.s.d.'s are taken into account individually in the estimation of e.s.d.'s in distances, angles and torsion angles; correlations between e.s.d.'s in cell parameters are only used when they are defined by crystal symmetry. An approximate (isotropic) treatment of cell e.s.d.'s is used for estimating e.s.d.'s involving l.s. planes.
Refinement. Refinement of *F*^2^ against ALL reflections. The weighted *R*-factor *wR* and goodness of fit *S* are based on *F*^2^, conventional *R*-factors *R* are based on *F*, with *F* set to zero for negative *F*^2^. The threshold expression of *F*^2^ > σ(*F*^2^) is used only for calculating *R*-factors(gt) *etc*. and is not relevant to the choice of reflections for refinement. *R*-factors based on *F*^2^ are statistically about twice as large as those based on *F*, and *R*- factors based on ALL data will be even larger.

### Fractional atomic coordinates and isotropic or equivalent isotropic displacement parameters (Å^2^)

**Table:**

	*x*	*y*	*z*	*U*_iso_*/*U*_eq_	Occ. (<1)
S1	0.56559 (11)	0.62819 (8)	0.28174 (3)	0.0203 (2)	
F1	0.6128 (3)	0.0664 (2)	0.52691 (10)	0.0370 (6)	
F2	0.4339 (3)	0.0220 (2)	0.57256 (9)	0.0375 (7)	
F3	0.5905 (3)	0.1477 (2)	0.59451 (8)	0.0375 (7)	
O1	0.4429 (3)	0.5738 (2)	0.25762 (10)	0.0264 (7)	
O2	0.7026 (3)	0.6337 (2)	0.25783 (10)	0.0255 (7)	
O3	0.3005 (3)	0.6299 (2)	0.35343 (10)	0.0281 (7)	
O4	0.1280 (3)	0.6875 (2)	0.29946 (10)	0.0309 (7)	
O5	0.7345 (3)	0.6836 (2)	0.37178 (10)	0.0247 (7)	
O6	0.5061 (3)	0.2805 (2)	0.51957 (9)	0.0231 (6)	
N1	0.5902 (4)	0.5577 (3)	0.33139 (11)	0.0212 (8)	
H1N	0.5523	0.4909	0.3326	0.025*	
N2	0.6642 (3)	0.5309 (3)	0.41268 (11)	0.0181 (7)	
H2N	0.7253	0.5502	0.4365	0.022*	
N3	0.5872 (3)	0.4023 (3)	0.46661 (11)	0.0189 (7)	
N4	0.4099 (3)	0.2653 (3)	0.44242 (11)	0.0176 (7)	
N5	0.4945 (3)	0.3976 (3)	0.38546 (11)	0.0184 (7)	
N6	0.3250 (4)	0.2637 (3)	0.36367 (11)	0.0205 (7)	
C1	0.5087 (4)	0.7661 (3)	0.29462 (13)	0.0221 (9)	
C2	0.3565 (4)	0.7803 (3)	0.30249 (13)	0.0225 (9)	
C3	0.2952 (5)	0.8858 (4)	0.29946 (14)	0.0280 (10)	
H3	0.1920	0.8964	0.3031	0.034*	
C4	0.3865 (5)	0.9744 (4)	0.29105 (15)	0.0317 (11)	
H4	0.3453	1.0462	0.2877	0.038*	
C5	0.5362 (5)	0.9600 (4)	0.28741 (14)	0.0303 (11)	
H5	0.5968	1.0234	0.2842	0.036*	
C6	0.6036 (5)	0.8560 (3)	0.28827 (14)	0.0238 (9)	
C7	0.2625 (4)	0.6893 (4)	0.32031 (14)	0.0239 (9)	
C8	0.0305 (5)	0.6065 (4)	0.31996 (17)	0.0349 (11)	
H8A	−0.0634	0.6049	0.3014	0.052*	
H8B	0.0119	0.6263	0.3534	0.052*	
H8C	0.0771	0.5336	0.3190	0.052*	
C9	0.7692 (5)	0.8509 (4)	0.28342 (16)	0.0312 (11)	
H9A	0.8117	0.7994	0.3072	0.047*	
H9B	0.8117	0.9244	0.2888	0.047*	
H9C	0.7919	0.8256	0.2510	0.047*	
C10	0.6673 (4)	0.5971 (3)	0.37173 (14)	0.0199 (9)	
C11	0.5788 (4)	0.4397 (3)	0.42095 (13)	0.0172 (8)	
C12	0.4983 (4)	0.3161 (3)	0.47348 (13)	0.0181 (9)	
C13	0.4114 (4)	0.3103 (3)	0.39785 (13)	0.0180 (8)	
C14	0.3193 (5)	0.3087 (4)	0.31491 (15)	0.0336 (11)	
H14A	0.4166	0.3010	0.3007	0.050*	
H14B	0.2923	0.3870	0.3160	0.050*	
H14C	0.2452	0.2684	0.2953	0.050*	
C15	0.2340 (5)	0.1684 (3)	0.37523 (15)	0.0239 (9)	
H15A	0.1771	0.1847	0.4038	0.036*	
H15B	0.2979	0.1044	0.3817	0.036*	
H15C	0.1660	0.1520	0.3480	0.036*	
C16	0.4144 (4)	0.1905 (3)	0.53311 (14)	0.0213 (9)	
H16A	0.3634	0.1582	0.5044	0.026*	
H16B	0.3386	0.2160	0.5555	0.026*	
C17	0.5130 (5)	0.1069 (3)	0.55699 (15)	0.0255 (10)	
O7	0.9666 (3)	0.7180 (2)	0.44998 (10)	0.0295 (7)	
N7	0.7687 (4)	0.4755 (3)	0.55253 (12)	0.0223 (8)	
H7NA	0.7109	0.4509	0.5288	0.027*	
H7NB	0.7634	0.4465	0.5814	0.027*	
N8	0.8653 (3)	0.5975 (3)	0.49936 (11)	0.0189 (7)	
N9	1.0539 (4)	0.7211 (3)	0.52842 (12)	0.0231 (8)	
N10	0.9501 (3)	0.5909 (3)	0.58237 (11)	0.0203 (8)	
N11	1.1295 (4)	0.7147 (3)	0.60813 (12)	0.0265 (8)	
C18	0.8641 (4)	0.5565 (3)	0.54471 (14)	0.0168 (8)	
C19	0.9634 (4)	0.6784 (3)	0.49539 (14)	0.0197 (9)	
C20	1.0409 (4)	0.6737 (3)	0.57225 (14)	0.0218 (9)	
C21	1.1218 (5)	0.6725 (4)	0.65693 (15)	0.0350 (11)	
H21A	1.0998	0.5934	0.6559	0.053*	
H21B	1.0434	0.7112	0.6737	0.053*	
H21C	1.2169	0.6845	0.6741	0.053*	
C22	1.2214 (5)	0.8123 (4)	0.60188 (17)	0.0310 (11)	
H22A	1.2314	0.8268	0.5675	0.046*	
H22B	1.3196	0.7996	0.6169	0.046*	
H22C	1.1751	0.8759	0.6170	0.046*	
C23	1.0678 (5)	0.8054 (4)	0.44104 (18)	0.0373 (12)	
H23A	1.1308	0.8188	0.4702	0.045*	
H23B	1.1329	0.7841	0.4146	0.045*	
C24	0.9862 (6)	0.9065 (5)	0.4312 (2)	0.0309 (14)	0.790 (5)
F4	1.0785 (5)	0.9900 (3)	0.42348 (16)	0.0455 (13)	0.790 (5)
F5	0.8890 (5)	0.9023 (4)	0.39341 (13)	0.0451 (13)	0.790 (5)
F6	0.9060 (4)	0.9328 (3)	0.46897 (14)	0.0463 (11)	0.790 (5)
C24'	1.0073 (19)	0.8858 (14)	0.4087 (6)	0.031*	0.210 (5)
F4'	1.089 (2)	0.9758 (16)	0.4046 (7)	0.046*	0.210 (5)
F5'	0.8679 (19)	0.9135 (19)	0.4108 (7)	0.046*	0.210 (5)
F6'	1.0249 (14)	0.8267 (10)	0.3691 (4)	0.046*	0.210 (5)

### Atomic displacement parameters (Å^2^)

**Table:**

	*U*^11^	*U*^22^	*U*^33^	*U*^12^	*U*^13^	*U*^23^
S1	0.0215 (5)	0.0214 (5)	0.0180 (5)	0.0006 (4)	−0.0005 (4)	0.0022 (4)
F1	0.0255 (13)	0.0324 (15)	0.0534 (17)	0.0003 (12)	0.0053 (12)	−0.0091 (13)
F2	0.0352 (15)	0.0279 (14)	0.0492 (16)	−0.0085 (12)	−0.0033 (12)	0.0154 (12)
F3	0.0422 (16)	0.0447 (16)	0.0247 (13)	−0.0129 (13)	−0.0143 (12)	0.0080 (12)
O1	0.0283 (16)	0.0277 (16)	0.0228 (15)	−0.0022 (13)	−0.0050 (12)	−0.0015 (13)
O2	0.0235 (15)	0.0285 (16)	0.0250 (15)	0.0012 (13)	0.0055 (12)	0.0008 (13)
O3	0.0240 (15)	0.0363 (17)	0.0241 (16)	−0.0005 (14)	0.0005 (13)	0.0098 (14)
O4	0.0246 (16)	0.0371 (18)	0.0306 (17)	0.0011 (14)	−0.0037 (13)	0.0042 (14)
O5	0.0268 (16)	0.0226 (16)	0.0246 (15)	−0.0058 (13)	−0.0023 (12)	0.0034 (13)
O6	0.0266 (15)	0.0256 (16)	0.0171 (14)	−0.0106 (13)	−0.0006 (12)	0.0057 (12)
N1	0.0266 (19)	0.0157 (17)	0.0210 (18)	−0.0041 (15)	−0.0029 (15)	0.0025 (14)
N2	0.0200 (17)	0.0176 (17)	0.0165 (16)	−0.0026 (14)	−0.0022 (14)	0.0013 (14)
N3	0.0203 (17)	0.0190 (17)	0.0174 (17)	−0.0013 (14)	−0.0002 (14)	0.0038 (14)
N4	0.0195 (17)	0.0161 (17)	0.0172 (17)	−0.0011 (14)	0.0018 (14)	0.0007 (14)
N5	0.0223 (18)	0.0149 (17)	0.0179 (17)	−0.0013 (14)	0.0012 (14)	−0.0004 (14)
N6	0.0205 (18)	0.0198 (18)	0.0209 (18)	−0.0043 (15)	−0.0040 (14)	−0.0010 (14)
C1	0.028 (2)	0.025 (2)	0.013 (2)	0.0004 (19)	−0.0004 (17)	0.0039 (17)
C2	0.027 (2)	0.029 (2)	0.0112 (19)	−0.0020 (19)	−0.0018 (17)	0.0025 (17)
C3	0.034 (2)	0.031 (3)	0.019 (2)	0.007 (2)	0.0013 (18)	0.0015 (19)
C4	0.045 (3)	0.026 (2)	0.024 (2)	0.013 (2)	0.007 (2)	0.0048 (19)
C5	0.047 (3)	0.023 (2)	0.021 (2)	−0.002 (2)	0.003 (2)	0.0041 (18)
C6	0.030 (2)	0.027 (2)	0.015 (2)	−0.0036 (19)	−0.0035 (17)	0.0028 (17)
C7	0.021 (2)	0.030 (2)	0.020 (2)	0.0044 (19)	0.0001 (17)	−0.0036 (19)
C8	0.021 (2)	0.040 (3)	0.043 (3)	−0.006 (2)	0.000 (2)	0.005 (2)
C9	0.034 (3)	0.030 (3)	0.029 (2)	−0.008 (2)	−0.003 (2)	0.005 (2)
C10	0.018 (2)	0.022 (2)	0.020 (2)	0.0035 (18)	0.0022 (17)	0.0001 (17)
C11	0.017 (2)	0.017 (2)	0.018 (2)	0.0030 (17)	0.0025 (16)	0.0015 (16)
C12	0.020 (2)	0.020 (2)	0.014 (2)	0.0003 (18)	0.0012 (16)	0.0016 (16)
C13	0.019 (2)	0.017 (2)	0.018 (2)	0.0030 (17)	0.0052 (16)	0.0007 (17)
C14	0.046 (3)	0.034 (3)	0.020 (2)	−0.009 (2)	−0.008 (2)	0.003 (2)
C15	0.026 (2)	0.020 (2)	0.026 (2)	−0.0012 (18)	0.0015 (18)	−0.0030 (18)
C16	0.020 (2)	0.023 (2)	0.021 (2)	−0.0070 (18)	0.0007 (17)	0.0032 (18)
C17	0.028 (2)	0.024 (2)	0.024 (2)	−0.0104 (19)	−0.0005 (19)	0.0041 (19)
O7	0.0242 (16)	0.0336 (17)	0.0304 (17)	−0.0128 (13)	−0.0059 (13)	0.0152 (14)
N7	0.0244 (18)	0.0258 (19)	0.0164 (17)	−0.0059 (16)	−0.0016 (14)	0.0028 (15)
N8	0.0188 (17)	0.0189 (17)	0.0191 (17)	0.0002 (14)	0.0009 (14)	0.0020 (14)
N9	0.0217 (18)	0.0203 (18)	0.0269 (19)	−0.0009 (15)	−0.0048 (15)	0.0023 (15)
N10	0.0207 (18)	0.0220 (19)	0.0183 (17)	−0.0004 (15)	0.0006 (14)	−0.0030 (14)
N11	0.0265 (19)	0.027 (2)	0.0258 (19)	−0.0064 (16)	−0.0035 (15)	−0.0050 (16)
C18	0.0152 (19)	0.015 (2)	0.020 (2)	0.0055 (16)	0.0003 (16)	−0.0014 (16)
C19	0.018 (2)	0.017 (2)	0.024 (2)	−0.0020 (17)	0.0012 (17)	0.0020 (17)
C20	0.020 (2)	0.021 (2)	0.023 (2)	0.0029 (18)	0.0002 (18)	−0.0043 (18)
C21	0.040 (3)	0.039 (3)	0.026 (2)	−0.009 (2)	−0.005 (2)	−0.005 (2)
C22	0.027 (2)	0.024 (2)	0.041 (3)	−0.006 (2)	−0.008 (2)	−0.006 (2)
C23	0.030 (3)	0.037 (3)	0.044 (3)	−0.014 (2)	−0.004 (2)	0.016 (2)
C24	0.028 (3)	0.026 (3)	0.038 (4)	−0.012 (3)	−0.006 (3)	0.009 (3)
F4	0.039 (2)	0.027 (2)	0.071 (3)	−0.0163 (18)	0.002 (2)	0.017 (2)
F5	0.051 (3)	0.038 (2)	0.044 (3)	−0.0095 (19)	−0.024 (2)	0.017 (2)
F6	0.037 (2)	0.033 (2)	0.069 (3)	−0.0029 (16)	0.0150 (19)	−0.0078 (18)

### Geometric parameters (Å, °)

**Table:**

S1—O2	1.426 (3)	C9—H9B	0.9800
S1—O1	1.438 (3)	C9—H9C	0.9800
S1—N1	1.633 (3)	C14—H14A	0.9800
S1—C1	1.789 (4)	C14—H14B	0.9800
F1—C17	1.343 (5)	C14—H14C	0.9800
F2—C17	1.334 (5)	C15—H15A	0.9800
F3—C17	1.333 (5)	C15—H15B	0.9800
O3—C7	1.211 (5)	C15—H15C	0.9800
O4—C7	1.329 (5)	C16—C17	1.491 (6)
O4—C8	1.448 (5)	C16—H16A	0.9900
O5—C10	1.213 (5)	C16—H16B	0.9900
O6—C12	1.353 (4)	O7—C19	1.353 (5)
O6—C16	1.428 (5)	O7—C23	1.427 (5)
N1—C10	1.386 (5)	N7—C18	1.329 (5)
N1—H1N	0.8800	N7—H7NA	0.8800
N2—C11	1.371 (5)	N7—H7NB	0.8800
N2—C10	1.395 (5)	N8—C19	1.329 (5)
N2—H2N	0.8800	N8—C18	1.357 (5)
N3—C12	1.336 (5)	N9—C19	1.316 (5)
N3—C11	1.349 (5)	N9—C20	1.356 (5)
N4—C12	1.311 (5)	N10—C20	1.332 (5)
N4—C13	1.355 (5)	N10—C18	1.350 (5)
N5—C11	1.329 (5)	N11—C20	1.354 (5)
N5—C13	1.349 (5)	N11—C21	1.454 (5)
N6—C13	1.335 (5)	N11—C22	1.459 (5)
N6—C15	1.461 (5)	C21—H21A	0.9800
N6—C14	1.461 (5)	C21—H21B	0.9800
C1—C6	1.402 (6)	C21—H21C	0.9800
C1—C2	1.410 (6)	C22—H22A	0.9800
C2—C3	1.394 (6)	C22—H22B	0.9800
C2—C7	1.488 (6)	C22—H22C	0.9800
C3—C4	1.379 (6)	C23—C24'	1.422 (18)
C3—H3	0.9500	C23—C24	1.451 (7)
C4—C5	1.371 (6)	C23—H23A	0.9900
C4—H4	0.9500	C23—H23B	0.9900
C5—C6	1.400 (6)	C24—F4	1.334 (6)
C5—H5	0.9500	C24—F6	1.335 (7)
C6—C9	1.509 (6)	C24—F5	1.349 (6)
C8—H8A	0.9800	C24'—F5'	1.307 (14)
C8—H8B	0.9800	C24'—F4'	1.322 (14)
C8—H8C	0.9800	C24'—F6'	1.330 (14)
C9—H9A	0.9800		
			
O2—S1—O1	118.18 (17)	N6—C15—H15A	109.5
O2—S1—N1	108.84 (17)	N6—C15—H15B	109.5
O1—S1—N1	103.68 (17)	H15A—C15—H15B	109.5
O2—S1—C1	108.01 (18)	N6—C15—H15C	109.5
O1—S1—C1	107.44 (18)	H15A—C15—H15C	109.5
N1—S1—C1	110.57 (17)	H15B—C15—H15C	109.5
C7—O4—C8	113.5 (3)	O6—C16—C17	107.0 (3)
C12—O6—C16	118.8 (3)	O6—C16—H16A	110.3
C10—N1—S1	123.8 (3)	C17—C16—H16A	110.3
C10—N1—H1N	118.1	O6—C16—H16B	110.3
S1—N1—H1N	118.1	C17—C16—H16B	110.3
C11—N2—C10	128.8 (3)	H16A—C16—H16B	108.6
C11—N2—H2N	115.6	F3—C17—F2	107.8 (3)
C10—N2—H2N	115.6	F3—C17—F1	106.1 (3)
C12—N3—C11	112.5 (3)	F2—C17—F1	107.4 (3)
C12—N4—C13	113.0 (3)	F3—C17—C16	112.8 (3)
C11—N5—C13	114.9 (3)	F2—C17—C16	110.5 (3)
C13—N6—C15	119.9 (3)	F1—C17—C16	112.0 (3)
C13—N6—C14	120.4 (3)	C19—O7—C23	117.4 (3)
C15—N6—C14	119.8 (3)	C18—N7—H7NA	120.0
C6—C1—C2	121.9 (4)	C18—N7—H7NB	120.0
C6—C1—S1	121.3 (3)	H7NA—N7—H7NB	120.0
C2—C1—S1	115.7 (3)	C19—N8—C18	111.9 (3)
C3—C2—C1	119.4 (4)	C19—N9—C20	113.0 (3)
C3—C2—C7	118.0 (4)	C20—N10—C18	114.3 (3)
C1—C2—C7	122.1 (4)	C20—N11—C21	120.9 (4)
C4—C3—C2	119.0 (4)	C20—N11—C22	122.3 (4)
C4—C3—H3	120.5	C21—N11—C22	116.2 (3)
C2—C3—H3	120.5	N7—C18—N10	117.5 (3)
C5—C4—C3	120.8 (4)	N7—C18—N8	116.6 (3)
C5—C4—H4	119.6	N10—C18—N8	125.9 (4)
C3—C4—H4	119.6	N9—C19—N8	129.3 (4)
C4—C5—C6	122.9 (4)	N9—C19—O7	118.7 (3)
C4—C5—H5	118.5	N8—C19—O7	112.0 (3)
C6—C5—H5	118.5	N10—C20—N11	118.3 (4)
C5—C6—C1	115.7 (4)	N10—C20—N9	125.5 (4)
C5—C6—C9	117.9 (4)	N11—C20—N9	116.2 (4)
C1—C6—C9	126.4 (4)	N11—C21—H21A	109.5
O3—C7—O4	123.7 (4)	N11—C21—H21B	109.5
O3—C7—C2	122.9 (4)	H21A—C21—H21B	109.5
O4—C7—C2	113.0 (4)	N11—C21—H21C	109.5
O4—C8—H8A	109.5	H21A—C21—H21C	109.5
O4—C8—H8B	109.5	H21B—C21—H21C	109.5
H8A—C8—H8B	109.5	N11—C22—H22A	109.5
O4—C8—H8C	109.5	N11—C22—H22B	109.5
H8A—C8—H8C	109.5	H22A—C22—H22B	109.5
H8B—C8—H8C	109.5	N11—C22—H22C	109.5
C6—C9—H9A	109.5	H22A—C22—H22C	109.5
C6—C9—H9B	109.5	H22B—C22—H22C	109.5
H9A—C9—H9B	109.5	O7—C23—C24	109.6 (4)
C6—C9—H9C	109.5	O7—C23—H23A	109.5
H9A—C9—H9C	109.5	C24—C23—H23A	107.0
H9B—C9—H9C	109.5	O7—C23—H23B	109.6
O5—C10—N1	122.5 (4)	C24—C23—H23B	113.0
O5—C10—N2	121.4 (4)	H23A—C23—H23B	108.1
N1—C10—N2	116.1 (3)	C24'—C23—O7	112.5 (8)
N5—C11—N3	125.7 (4)	C24'—C23—H23A	127.2
N5—C11—N2	119.8 (3)	C24'—C23—H23B	86.3
N3—C11—N2	114.5 (3)	F4—C24—F6	107.7 (5)
N4—C12—N3	129.0 (3)	F4—C24—F5	107.3 (4)
N4—C12—O6	119.1 (3)	F6—C24—F5	105.4 (5)
N3—C12—O6	111.8 (3)	F4—C24—C23	110.7 (5)
N6—C13—N5	117.8 (3)	F6—C24—C23	109.8 (4)
N6—C13—N4	117.3 (3)	F5—C24—C23	115.4 (5)
N5—C13—N4	124.9 (3)	F5'—C24'—F4'	109.3 (16)
N6—C14—H14A	109.5	F5'—C24'—F6'	108.4 (15)
N6—C14—H14B	109.5	F4'—C24'—F6'	106.9 (14)
H14A—C14—H14B	109.5	F5'—C24'—C23	120.0 (17)
N6—C14—H14C	109.5	F4'—C24'—C23	114.6 (16)
H14A—C14—H14C	109.5	F6'—C24'—C23	95.8 (12)
H14B—C14—H14C	109.5		
			
O2—S1—N1—C10	72.3 (3)	C16—O6—C12—N4	−1.8 (5)
O1—S1—N1—C10	−161.1 (3)	C16—O6—C12—N3	178.3 (3)
C1—S1—N1—C10	−46.2 (4)	C15—N6—C13—N5	179.5 (3)
O2—S1—C1—C6	−9.8 (4)	C14—N6—C13—N5	−1.5 (5)
O1—S1—C1—C6	−138.3 (3)	C15—N6—C13—N4	0.1 (5)
N1—S1—C1—C6	109.2 (3)	C14—N6—C13—N4	179.1 (4)
O2—S1—C1—C2	158.7 (3)	C11—N5—C13—N6	−179.5 (3)
O1—S1—C1—C2	30.2 (3)	C11—N5—C13—N4	−0.2 (5)
N1—S1—C1—C2	−82.3 (3)	C12—N4—C13—N6	179.8 (3)
C6—C1—C2—C3	6.1 (6)	C12—N4—C13—N5	0.4 (5)
S1—C1—C2—C3	−162.3 (3)	C12—O6—C16—C17	127.5 (4)
C6—C1—C2—C7	−165.6 (4)	O6—C16—C17—F3	56.6 (4)
S1—C1—C2—C7	26.0 (5)	O6—C16—C17—F2	177.4 (3)
C1—C2—C3—C4	−3.0 (6)	O6—C16—C17—F1	−63.0 (4)
C7—C2—C3—C4	169.0 (4)	C20—N10—C18—N7	−178.7 (3)
C2—C3—C4—C5	−2.3 (6)	C20—N10—C18—N8	0.9 (5)
C3—C4—C5—C6	4.9 (7)	C19—N8—C18—N7	179.5 (3)
C4—C5—C6—C1	−1.9 (6)	C19—N8—C18—N10	0.0 (5)
C4—C5—C6—C9	179.4 (4)	C20—N9—C19—N8	−0.4 (6)
C2—C1—C6—C5	−3.6 (6)	C20—N9—C19—O7	−179.4 (3)
S1—C1—C6—C5	164.1 (3)	C18—N8—C19—N9	−0.2 (6)
C2—C1—C6—C9	175.0 (4)	C18—N8—C19—O7	178.9 (3)
S1—C1—C6—C9	−17.2 (6)	C23—O7—C19—N9	−0.8 (5)
C8—O4—C7—O3	−0.4 (6)	C23—O7—C19—N8	−180.0 (4)
C8—O4—C7—C2	−174.0 (3)	C18—N10—C20—N11	179.0 (3)
C3—C2—C7—O3	−126.3 (4)	C18—N10—C20—N9	−1.6 (6)
C1—C2—C7—O3	45.5 (6)	C21—N11—C20—N10	−2.4 (6)
C3—C2—C7—O4	47.4 (5)	C22—N11—C20—N10	−173.6 (4)
C1—C2—C7—O4	−140.8 (4)	C21—N11—C20—N9	178.1 (4)
S1—N1—C10—O5	−7.0 (5)	C22—N11—C20—N9	6.9 (6)
S1—N1—C10—N2	173.4 (3)	C19—N9—C20—N10	1.4 (6)
C11—N2—C10—O5	170.2 (4)	C19—N9—C20—N11	−179.2 (3)
C11—N2—C10—N1	−10.2 (6)	C19—O7—C23—C24	−110.7 (4)
C13—N5—C11—N3	0.3 (5)	O7—C23—C24—F4	179.0 (4)
C13—N5—C11—N2	−177.9 (3)	O7—C23—C24—F6	60.1 (5)
C12—N3—C11—N5	−0.7 (5)	O7—C23—C24—F5	−58.8 (6)
C12—N3—C11—N2	177.6 (3)	C19—O7—C23—C24'	−141.0 (8)
C10—N2—C11—N5	6.9 (6)	O7—C23—C24'—F5'	37.7 (17)
C10—N2—C11—N3	−171.5 (4)	C24—C23—C24'—F5'	−52.0 (16)
C13—N4—C12—N3	−0.9 (6)	O7—C23—C24'—F4'	171.0 (11)
C13—N4—C12—O6	179.2 (3)	C24—C23—C24'—F4'	81.3 (18)
C11—N3—C12—N4	1.1 (6)	O7—C23—C24'—F6'	−77.5 (11)
C11—N3—C12—O6	−179.1 (3)	C24—C23—C24'—F6'	−167 (2)

### Hydrogen-bond geometry (Å, °)

**Table:**

*D*—H···*A*	*D*—H	H···*A*	*D*···*A*	*D*—H···*A*
N1—H1N···N5	0.88	1.94	2.620 (4)	133.
N2—H2N···N8	0.88	2.20	3.080 (4)	176.
N7—H7NA···N3	0.88	2.11	2.989 (5)	174.
N7—H7NB···O3^i^	0.88	2.13	2.996 (4)	167.
C5—H5···O2^ii^	0.95	2.57	3.434 (5)	152.
C8—H8A···O2^iii^	0.98	2.43	3.398 (5)	170.
C9—H9A···O5	0.98	2.40	3.210 (5)	140.
C16—H16B···O5^i^	0.99	2.47	3.373 (5)	152.

Symmetry codes: (i) −*x*+1, −*y*+1, −*z*+1; (ii) −*x*+3/2, *y*+1/2, −*z*+1/2; (iii) *x*−1, *y*, *z*.
